# The hexane fraction of *Ardisia crispa* Thunb. A. DC. roots inhibits inflammation-induced angiogenesis

**DOI:** 10.1186/1472-6882-13-5

**Published:** 2013-01-08

**Authors:** Dayang Erna Zulaikha Awang Hamsin, Roslida Abdul Hamid, Latifah Saiful Yazan, Che Norma Mat Taib, Yeong Looi Ting

**Affiliations:** 1 Department of Biomedical Science, Faculty of Medicine and Health Sciences, Universiti Putra Malaysia, Serdang, Selangor, 43400, Malaysia; 2 Department of Anatomy, Faculty of Medicine and Health Sciences, Universiti Putra Malaysia, Serdang, Selangor, 43400, Malaysia; 3 Department of Paraclinical Science, Faculty of Medicine and Health Sciences, Universiti Malaysia Sarawak, Kuching, Sarawak, 93150, Malaysia

**Keywords:** *Ardisia crispa*, Miles vascular permeability assay, Murine air pouch granuloma, Angiogenesis

## Abstract

**Background:**

*Ardisia crispa* (Myrsinaceae) is used in traditional Malay medicine to treat various ailments associated with inflammation, including rheumatism. The plant’s hexane fraction was previously shown to inhibit several diseases associated with inflammation. As there is a strong correlation between inflammation and angiogenesis, we conducted the present study to investigate the anti-angiogenic effects of the plant’s roots in animal models of inflammation-induced angiogenesis.

**Methods:**

We first performed phytochemical screening and high-performance liquid chromatography (HPLC) fingerprinting of the hexane fraction of *Ardisia crispa* roots ethanolic extract (ACRH) and its quinone-rich fraction (QRF). The anti-inflammatory properties of ACRH and QRF were tested using the Miles vascular permeability assay and the murine air pouch granuloma model following oral administration at various doses.

**Results:**

Preliminary phytochemical screening of ACRH revealed the presence of flavonoids, triterpenes, and tannins. The QRF was separated from ACRH (38.38% w/w) by column chromatography, and was isolated to yield a benzoquinonoid compound. The ACRH and QRF were quantified by HPLC. The LD_50_ value of ACRH was 617.02 mg/kg. In the Miles vascular permeability assay, the lowest dose of ACRH (10 mg/kg) and all doses of QRF significantly reduced vascular endothelial growth factor (VEGF)-induced hyperpermeability, when compared with the vehicle control. In the murine air pouch granuloma model, ACRH and QRF both displayed significant and dose-dependent anti-inflammatory effects, without granuloma weight. ACRH and QRF significantly reduced the vascular index, but not granuloma tissue weight.

**Conclusions:**

In conclusion, both ACRH and QRF showed potential anti-inflammatory properties in a model of inflammation-induced angiogenesis model, demonstrating their potential anti-angiogenic properties.

## Background

Angiogenesis is the process of blood vessel formation. It is important in normal physiology and is involved in the progression of chronic diseases, particularly cancer, arthritis and cardiovascular diseases [1-7]. The advantage of targeting angiogenesis to control pathological disorders, particularly cancer, is that anti-angiogenic agents specifically target newly formed blood vessels without damaging existing blood vessels [1]. The important role of angiogenesis in the pathogenesis of chronic inflammatory diseases has led to the implementation of anti-angiogenic strategies for treating these diseases [8].

Drugs are increasingly being developed from natural products, offering a very promising approach to identify novel anti-angiogenic and anti-cancer agents [9]. A considerable number of bioactive compounds derived from functional and medicinal foods have been identified as potential anti-angiogenic agents based on the results of experimental and clinical studies. Compounds such as curcumin in turmeric [10], naringenin in citrus [11], humulone in beer hop [12], betulinic acid in almond hull [13], capsaicin in pepper [14] and resveratrol in grapes [15,16] were reported to inhibit angiogenesis by targeting the cyclooxygenase-2 (COX-2) and 5-lipoxygenase (5-LOX) pathways. Inhibition of the COX-2 pathway, for example, was shown to reduce prostaglandin E_2_ (PGE_2_) production [17], which may suppress vascular endothelial growth factor (VEGF) expression [18], a key mediator of *in vitro* angiogenesis [19].

*Ardisia crispa* Thunb A.DC is an evergreen shrub belonging to Myrsinaceae family, and is widely distributed and indigenous throughout Asia. In Malaysia, *Ardisia crispa* can be found in the undergrowth of jungle peripheries, in dappled shade, and in shady regions [20]. In Peninsular Malaysia, it is locally known as “Mata Ayam” or “Mata Itik”. The roots and leaves of the plant have been used by local villagers to treat various ailments as part of local traditional medicine. The roots were used as an ingredient to treat postnatal syndromes, while its boiled concoction was used to treating throat and chest discomfort, and rheumatism. The root’s juice was also reported to be useful for treating earache, cough, fever, and diarrhoea [21].

**Figure 1:**
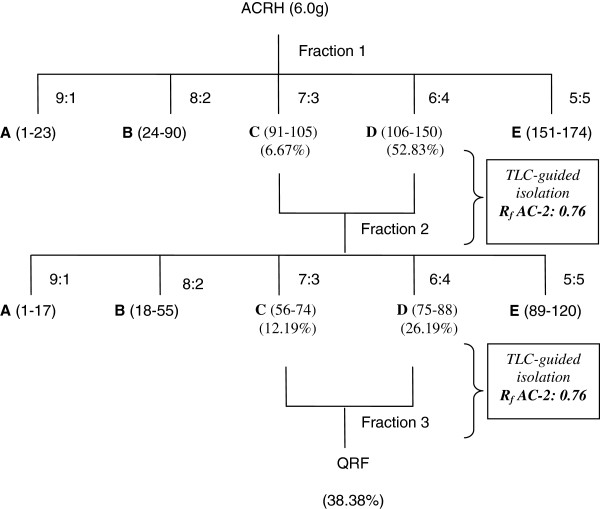
**Chromatographic separation and fractionation of *Ardisia crispa *roots.** Column chromatographic separation was repeated three times to collect QRF (38.38% w/w). **A–E** represent the five major fractions obtained using the hexane:ethyl acetate gradients. Fraction 1: **AC**-2 Rf 0.76 in fractions **C** and **D** (vials 91–150). The mixture of fractions **C** and **D** obtained in fraction 1 was separated to yield fraction 2. Fraction 2: **AC**-2 Rf 0.76 in fractions **C** and **D** (vials 56–88). The mixture of **C** and **D** from fraction 2 was separated again to yield QRF.

Phytochemical studies reported that several pharmacologically active compounds, including triterpenoid saponins [22], n-peptide [23], and benzoquinone [24] were isolated from *Ardisia crispa*. Meanwhile, its hexane fraction was reported to have anti-ulcerogenic [25], as well as having anti-inflammatory and anti-hyperalgesic properties *in vivo*[26]. Additionally, a benzoquinonoid compound, 2-methoxy-6-undercyl-1,4-benzoquinone (Figure 1), was isolated from the hexane fraction of *Ardisia crispa* roots and exhi-bited anti-inflammatory properties *in vivo*[27].

Angiogenesis plays an essential role in the initiation and progression of chronic inflammatory disorders [28]. It was proposed that angiogenesis is closely linked to inflammation in that they share similar activating pathways, including COX-2 [29,30]. Pro-inflammatory mediators, such as PGE_2_, were shown to induce angiogenesis *in vivo*[31,32], partly by increasing the expression of VEGF, a potent angiogenic factor [33,34]. Therefore, while *Ardisia crispa* roots were previously demonstrated to possess anti-inflammatory properties, it seems feasible that *Ardisia crispa* roots might also have anti-angiogenic properties, based on the significant correlation between inflammation and angiogenesis shown in previous reports [28,35]. Therefore, we conducted this study to explore the potential anti-angiogenic properties of *Ardisia crispa* roots. It is hoped that the findings from this study would provide a scientific basis to carry out further studies on the anti-angiogenic properties of *Ardisia crispa* roots.

## Methods

### Chemicals and drugs

Silica gel 60 (70–230 mesh), thin-layer chromatography (TLC) silica gel (60 F_254_ 0.25 mm), methanol and chloroform were purchased from Merck (Munchen, Germany). Trifluoroacetic acid (TFA) was purchased from Alfa Aesar (Wardhill, MA, USA). Phosphate-buffered saline (PBS), VEGF 165, carboxymethylcellulose (CMC), Evans Blue dye, formamide, carmine red, papain 12U/mL, and EDTA, *N*-acetylcysteine and indomethacin (purity > 99%) were purchased from Sigma (St. Louis, MO, USA). All other reagents were of analytical grade and were purchased from commercial sources.

### Plant material

*Ardisia crispa* roots (ACR) were collected in Machang (Kelantan, Malaysia) in April 2010, and were identified by Dr. Roslida Abdul Hamid (Universiti Putra Malaysia). A voucher specimen (20841) was deposited at the Herbarium of Universiti Kebangsaan Malaysia. ACR was dried in an oven for 5 days at 60°C (Memmert, Germany). The roots were then pulverised into coarse powder by using a mill grinder (Retsch, Germany). Compounds were extracted from ACR using the Soxhlet method. Briefly, 600 g of powdered ACR was extracted using 80% ethanol at a temperature of 80–90°C under reflux (3 × 2000 mL, 72 h each), then filtered and concentrated using rotary vacuum evaporator to yield *Ardisia crispa* ethanolic extract (ACRE). The ACRE was then fractionated using absolute *n*-hexane (3 × 1000 mL, 72 h each). The filtrate was concentrated under reduced pressure, yielding the *Ardisia crispa* root hexane fraction (ACRH).

### Phytochemical analysis

Preliminary phytochemical analysis of ACRH was carried out to detect the presence of alkaloids, saponin, flavonoids, tannins, polyphenolic compounds, triterpenes and steroids, using a previously described method [36].

### Separation of the quinone-rich fraction (QRF)

**Figure 2:**
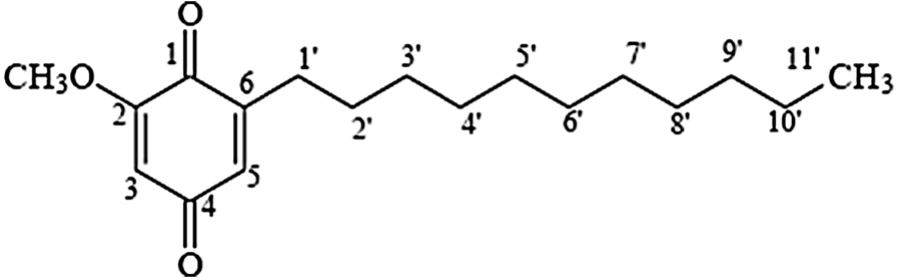
Chemical structure of 2-methoxy-6-undecyl-1,4-benzoquinone (AC-2).

Approximately 6 g of ACRH were loaded through a column (65 cm × 2 cm) packed with 200 g of 70–230 mesh silica gel (Merck). The sample was eluted with a mobile phase consisting of hexane:ethyl acetate (9:1–5:5 [v/v], 500 mL) at a constant flow rate. The eluents were collected in 20-mL fractions (Figure 1). TLC was used to qualitatively identify the presence of compounds by comparing the retention factor (R_f_) of individual compounds in each fraction relative to those of reference compounds. Chloroform was used as the developing solvent. Compounds were detected under an UV lamp at a wavelength of 254/356 nm (Mineralight®) and by heating the TLC plates with 10% sulphuric acid (H_2_SO_4_). Vials containing compounds with similar R_f_ values under H_2_SO_4_ were combined together to obtain the QRF. The R_f_ of this fraction was compared with that of the pure compound, AC-2, as obtained in a previous study [27]. AC-2, isolated from the *Ardisia crispa* roots, was provided by Dr Roslida Abdul Hamid. The compound was previously characterised by gas chromatography/mass spectrometry and nuclear magnetic resonance, and was identified as 2-methoxy-6-undecyl-1,4-benzoquinone (Figure 2) [27].

### HPLC analysis of QRF

We performed HPLC using the method reported by Shelar et al. [37], with slight modifications. The HPLC system consisted of an Agilent 1290 series Ultra Performance Liquid Chromatography (UPLC) equipped with an auto sampler, a diode array detector (DAD) and a reverse-phase analytical column (HyperClone 3u ODS C18, 3 μm, 4.0 × 125 mm; Phenomenex, USA). The mobile phase consisted of methanol (A) and 0.1% TFA (B) (88:12 [v/v]), and was degassed before use. The flow-rate was kept at 1.0 mL/min. The temperature of column was held at 29.99 ± 1°C. The injection volume was 20 μL. Detection was carried out at 288 nm.

### Experimental animals

Male ICR mice (25–30 g) at 6–8 weeks of age were used in this study. Six mice were housed per cage under standard laboratory conditions (temperature 25 ± 2°C and 12-h light/dark cycle). The animals had free access to water and food pellets *ad libitum*. Mice were acclimatised for 1 week before experiments. Ethical clearance was obtained from the Animal Care and Use Committee (ACUC), Faculty of Medicine and Health Sciences, Universiti Putra Malaysia, Malaysia (Reference number: UPM/FPSK/PADS/UUH/F05).

### Acute oral toxicity

ICR mice were allotted into groups of 10 animals each and were divided into seven different groups to assess the acute toxicity of ACRH, as previously described [38] ACRH was administered orally at single doses of 300, 400, 500, 800, 1200, 1600 and 1800 mg/kg. The time of administration was recorded and the mice were carefully examined for any clinical symptoms and abnormal behaviour. The number of deaths and toxicity signs in each group were recorded for 48 h. The proportion of mice that died in each group was calculated and plotted against the dose to determine the median lethal dose (LD_50_).

### Miles vascular permeability test

The vascular permeability assay was carried out according to the method of Pakhneshan et al. [39] with some modifications. Eight groups of six randomly selected mice per group were orally administered with a vehicle control (5% CMC), a positive control (10 mg/kg indomethacin), or with 10, 30, or 100 mg/kg of ACRH or QRF every day for 5 days. On day 6, the mice were first anesthetised with an intraperitoneal injection of Avertin mixture (10 mL/kg). Next, 100 μL of Evans blue (1% in 0.9% saline) was injected intravenously via a lateral tail vein. After 10 min, 50 μL of VEGF (1 ng/μL) was injected intradermally into the dorsal area. After 20 min, the mice were euthanised and a skin sample (1 cm^2^) was harvested, weighed, and incubated at 56°C for 24 h. The skin samples were then extracted in 1 mL formamide for 5 days at room temperature. The optical density of the formamide extract was determined at 620 nm (Shimadzu, Japan).

### Murine air pouch granuloma

This method was carried out as previous described with some modifications [40]. A total of 48 mice was randomly divided into eight groups (*n* = 6 mice/group) and treated with the vehicle control (0.5% CMC), 10 mg/kg indomethacin, or 10, 30, or 100 mg/kg ACRH or QRF. Air pouches were induced on day 1 by injecting 5 mL of filtered air (0.2 μm mesh) into the dorsal region of the mice. On days 3 and 5, approximately 3 mL of filtered air was injected again to maintain the air pouch. On the following day, 0.2 mL of 0.1% croton oil in Freund’s Complete Adjuvant (FA; v/v) was injected into the air pouch cavity. After Freund’s Complete Adjuvant (FCA) injection, the specified treatments were administered orally for 1 week to conscious mice. After 1 week of treatment, vascular casts were prepared by intravenously injecting 1 mL of 10% carmine red in 5% gelatine solution into the tail vein. The granulomatous pouch lining was treated as previously described [41]. The animals were chilled at 2–4°C for 4 h before removing the granuloma tissues. The granuloma tissues were dried at 56°C for 48 h and weighed. The dried tissues were cut and digested in 2 mL of digestive juice (12,000 U/L papain, 1 mmol/L EDTA, and 0.33 g/L *N*-acetylcysteine in PBS, pH 7.0) for 48 h at 56°C. Subsequently, 0.2 mL 5 mol/l NaOH was added to neutralise carminic acid. The solution was centrifuged at 2000 × *g* for 5 minutes. The supernatant was then filtered using a 0.2 μm syringe filter and the optical density of the filtrate was measured at 540 nm. The vascular index, as an index of new formation of blood vessels in the granuloma tissue lining, was determined by dividing carmine dye content by dry granuloma tissue (mg/g) [41].

### Statistical analysis

All data are expressed as means ± standard error of the mean (SEM) (*n* = 6 mice/group). One-way analysis of variance followed by the least significance difference *post hoc* test was used for statistical analyses, and were performed using SPSS software (version 16).

## Results

### Plant extraction

Extraction of *Ardisia crispa* roots with aqueous 80% ethanol yielded approximately 81.16 g (13.53% w/w) of ACRE. Further fractionation with n-hexane yielded 21.01 g (25.87% w/w) of ACRH. Preliminary phytochemical screening of ACRH indicated the presence of flavonoids, triterpenes and tannins. Further separation of ACRH yielded about 2.3 g (38.38% w/w) of QRF.

### Chromatographic analysis of ACRH and QRF

Eluents collected from the chromatography column loaded with 6 g of ACRH were run in a mobile phase, consisting of a gradient solvent of n-hexane/ethyl acetate at the ratio of 7:3–6:4, to harvest fraction 2. Fraction 2 was further re-chromatographed at a similar ratio of n-hexane/ethyl acetate to yield fraction 3, which was labelled as QRF. Qualitative analysis of QRF, by TLC, revealed the presence of a compound with similar chemical characteristics to a benzoquinonoid compound (AC-2) along with a few other spots. The QRF had a similar R_f_ to AC-2 (R_f_ = 0.76), which was detected as a pink spot at 254 nm, and turned black after being sprayed with H_2_SO_4_ and heated.

**Figure 3:**
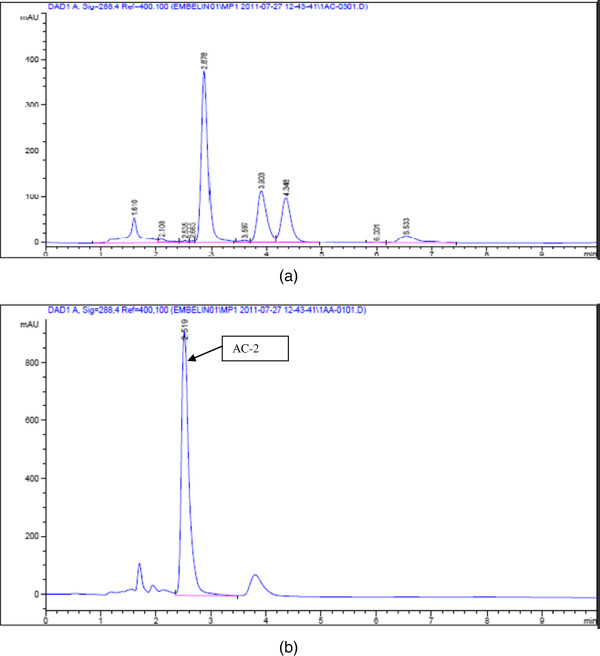
**High performance liquid chromatography (HPLC) fingerprints of QRF (a) and AC-2 (b).** R_t_ for AC_2_ = 2.878 min. HPLC fingerprint of another benzoquinonoid compound isolated from QRF in *Ardisia crispa* roots (R_t_ = 2.619 min).

An HPLC-based method was successfully applied using a reverse-phase column with UV detection at 288 nm using HPLC-grade methanol as the mobile phase. This method resolved three major peaks for QRF (Figure 3a). The HPLC spectra for QRF showed major peaks at the retention times (in min) of 2.878, 3.903 and 4.348 at a wavelength of 288 nm. The major peak at R_t_ = 2.878 appeared to be AC-2 (2-methoxy-6-undecyl-1,4-benzoquinone) when compared with the spectrum for the reference compound (Figure 3b).

### Oral acute toxicity

LD_50_ was determined from the mortality curve and the LD_50_ of ACRH was found to be 617.02 mg/kg.

### Miles vascular permeability assay

**Figure 4:**
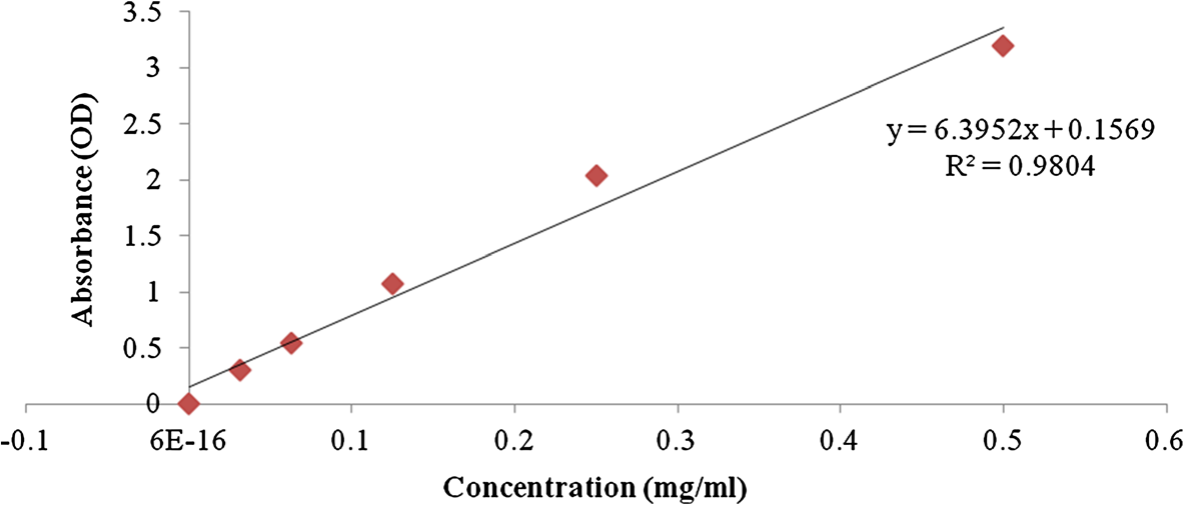
Standard curve for Evans Blue dye concentration (mg/mL) versus optical density.

In order to evaluate the anti-angiogenic potential of ACRH and QRF, we determined their inhibitory effects on vascular permeability. A standard curve of Evans blue dye concentration (mg/mL) versus absorbance (OD) was plotted (Figure 4). In the vehicle-treated group, the Evans blue dye concentration was 0.0597 ± 0.005 mg/mL. Administration of 10 mg/kg ACRH significantly reduced VEGF-induced vascular permeability with an Evans blue dye concentration of 0.0367 ± 0.008 mg/mL (*P* < 0.01), as compared with the vehicle control. However, ACRH at doses of 30 and 100 mg/kg did not have a significant inhibitory effect. QRF significantly reduced vascular permeability, as the Evans blue dye concentrations at doses of 10, 30 and 100 mg/kg were 0.0305 ± 0.005 mg/mL (*P* < 0.001), 0.0405 ± 0.006 (*P* < 0.05) and 0.0373 ± 0.005 (*P* < 0.01) respectively, when compared with the vehicle control (Table 1). The administration of 10 mg/kg indomethacin also significantly reduced the Evans blue dye concentration to 0.0340 ± 0.005 (*P* < 0.01), which is as equipotent to ACRH and the lower dose of QRF (10 mg/kg). The extent of vascular permeability inhibition, as a percentage of that in the control group, was 38.53%, 48.91% and 43.05% for ACRH, QRF and indomethacin, respectively, at doses of 10 mg/kg (Table 1).

**Table 1:** Evans blue dye concentrations and relative inhibition, as determined by the Miles vascular permeability test

**Treatment group**	**Dose (mg/kg)**	**Vascular Index (VI) (mg/g)**	**Inhibition (%)**
Vehicle control (CMC) 5%	-	0.0597 ± 0.005	-
Indomethacin	10	0.0340 ± 0.005^**^	43.05
ACRH	10	0.0367 ± 0.008^**^	38.53
	30	0.0495 ± 0.005	17.08
	100	0.0438 ± 0.007	26.63
QRF	10	0.0305 ± 0.005^***^	48.91
	30	0.0405 ± 0.006^*^	32.19
	100	0.0373 ± 0.005**	37.52

Data are means ± SEM (*n* = 6 mice/group). *P < 0.05, **P < 0.01 and ***P < 0.001 versus vehicle control.

### Murine air pouch granuloma

The inhibitory effects of ACRH and QRF on angiogenesis and inflammatory processes were evaluated by establishing adjuvant-induced air pouch granuloma in mice, and were measured in terms of the vascular index (VI) and granuloma tissue dry weight. In the vehicle control group, the VI was 1.599 ± 0.091 mg/g tissue weight. Administration of 30 and 100 mg/kg ACRH significantly reduced VI compared with the vehicle control, with values of 1.064 ± 0.126 and 0.912 ± 0.053 mg/g tissue weight, respectively, (both, *P* < 0.001). All three doses of QRF significantly reduced VI relative to the control group, with values of 0.956 ± 0.054, 0.586 ± 0.108 and 0.444 ± 0.051 mg/g tissue weight at doses of 10, 30 and 100 mg/kg, respectively (all, *P* < 0.001). Meanwhile, 10 mg/kg indomethacin significantly reduced VI to 1.028 ± 0.103 mg/g tissue weight (*P* < 0.001), and was equipotent to the lower dose of QRF. The inhibitory effects of ACRH and QRF on VI were dose-dependent (Table 2). The ED_50_ for QRF was 28.38 mg/g tissue weight.

**Table 2:** Effects of ACRH and QRF on the vascular index (VI) of air pouch granuloma

**Treatment group**	**Dose (mg/kg)**	**Vascular Index (VI) (mg/g)**	**Inhibition (%)**
Control (CMC)	0.5%	1.599 ± 0.091	-
Indomethacin	10	1.028 ± 0.103*	35.71
ACRH	10	1.366 ± 0.089	14.57
	30	1.064 ± 0.126*	33.45
	100	0.912 ± 0.053*	42.64
TQRF	10	0.956 ± 0.054*	40.21
	30	0.586 ± 0.108*	63.39
	100	0.444 ± 0.051*	72.23

Data are means ± SEM (n = 6 mice/group). VI was calculated as mg carmine dye content per gram tissue. *P < 0.001 versus vehicle control.

Interestingly, we found a different pattern of results in terms of granulomatous tissue dry weight, although there was still evidence for dose-dependent effects of ACRH and QRF. The granulomatous dry tissue weight in the vehicle control group was 0.446 ± 0.044 g, and was significantly reduced in the 10 mg/kg indomethacin group to 0.307 ± 0.011 g. Of note, only ACRH at 100 mg/kg significantly reduced granulomatous tissue dry weight relative to the control group, with a weight of 0.303 ± 0.024 g (*P* < 0.05). The decrease in granulomatous try tissue weight was not significant at any dose of QRF relative to the vehicle control group (Table 3). Thus, these results suggested that anti-angiogenic activity of QRF, as evidenced by the decrease in VI, did not result in marked reductions in weight of the granulomatous tissue.

**Table 3:** Effects of ACRH and QRF on the granulomatous tissue dry weight (g) in air pouch granuloma

**Treatment group**	**Dose (mg/kg)**	**Granuloma tissue dry weight (g)**	**Inhibition (%)**
Control (CMC)	0.5%	0.446 ± 0.044	-
Indomethacin	10	0.307 ± 0.011*	31.17
ACRH	10	0.511 ± 0.048	-
	30	0.346 ± 0.029	22.42
	100	0.303 ± 0.024*	32.06
TQRF	10	0.483 ± 0.056	-
	30	0.473 ± 0.040	-
	100	0.388 ± 0.019	-

Data are means ± SEM (*n* = 6). *P < 0.05 versus vehicle control.

## Discussion

*Ardisia* species are rich in polyphenols, triterpenoid saponins, isocoumarins, quinones and alkylphenols. *Ardisia* species and their constituents exhibit a wide range of biological activities, indicating that some of these plants could be exploited for the development of novel phytopharmaceuticals [42]. *Ardisia japonica* and *Ardisia sieboldii*, for example, were reported to inhibit 5-lipoxygenase [43,44], and therefore suppress inflammatory activities. Some *Ardisia* species were also reported to possess cytotoxic activities *in vitro*[45,46]. Because inhibition of angiogenesis has the potential to suppress tumour growth and metastases, its inhibition is one of the most promising strategies in the development of novel anti-cancer therapies, and in the treatment of other human diseases associated with angiogenesis. Thus far, however, no-one has reported on the anti-angiogenic properties of any *Ardisia* species, including *Ardisia crispa*.

Our preliminary phytochemical analysis of ACRH revealed that it contained significant amounts of triterpenes, flavonoids, and tannins. Although, the phytochemical screening did not reveal the presence of a benzoquinone, it was detected in the HPLC profiles of QRF when compared with the reference AC-2 compound, 2-methoxy-6-undecyl-1,4-benzoquinone. Benzoquinonoid isolated from *Ardisia crispa* was previously reported to possess anti-tumour and anti-metastatic properties *in vitro*[24].

The LD_50_ of ACRH was 617 mg/kg in the acute toxicity test. The doses of ACRH and QRF used in the experiments to determine their biological activities elicited no apparent behavioural side effects or signs of toxicity, such as convulsions. Nevertheless, a complete toxicity assessment of ACRH is needed to determine the margin of safety between the efficacy and toxicity of *Ardisia crispa* roots.

Several models are used to quantify angiogenesis and to evaluate the activities of candidate anti-angiogenic agents. These include *in vivo* models such as chorioallantoic membrane [47], rabbit cornea [48] and rat air pouch [49]. In the Miles vascular permeability assay, indomethacin was used as a positive control because it is an established anti-angiogenic agent, based on data obtained in the cornea and tumour [50]. Additionally, indomethacin was reported to strongly suppress VEGF-induced permeability in mice [39].

VEGF is a key factor in angiogenesis and vascular permeability, being involved in many pathological processes. VEGF-induced permeability in this assay was quantified by prior intravenous injection of Evans blue dye into the mice. Evans blue dye binds to plasma proteins and is a marker for vascular hyperpermeability to macromolecules. Increased permeability is measured by spectrophotometric quantification of the blue dye [51]. In the Miles assay, both ACRH and QRF significantly reduced VEGF-induced permeability. Interestingly, they had a therapeutic effect at the lowest dose tested (10 mg/kg), and that dose was more potent than the higher doses (30 and 100 mg/kg). The results obtained are consistent with previous reports, in which the anti-angiogenic therapies were more effective when administered at a more frequent, or metronomic, low-dose schedule [52]. It is postulated that the benzoquinonoid present in QRF might be responsible for the suppression of VEGF-induced permeability. Interestingly, a similar benzoquinonoid compound present in *A*. *crispa* was previously reported to possess anti-metastatic activity [24].

In this assay, vascular leakiness of the Evans blue dye was observed at sites of intradermal injection of VEGF, indicating increased vascular permeability. VEGF is a potent permeabilizing mediator that is essential for neovascularisation [53]. Suppression of VEGF reduces vascular leakiness and ultimately prevents the migration of plasma proteins, endothelial cells and fibrin rich matrix that are essential for neovascularisation [54]. Because it was previously reported that ACRH possesses anti-inflammatory properties by blocking COX-2 [27], the mechanism involved in the attenuation of vascular permeability might involve reduced synthesis of COX-2-derived prostanoids, including PGs. Increased vascular permeability was reported to be elicited by PGs through synergistic effects with other mediators [55]. PGs also contribute to VEGF-induced hyperpermeability by targeting vascular endothelial cells [56]. The mechanism by which PGE_2_ induces angiogenesis in this study is not fully understood. However, we can speculate that ACRH and QRF can halt neovascularisation by suppressing VEGF-induced vascular permeability.

Murine air pouch granuloma is an animal model of inflammation that is characterised by intense angiogenesis during the chronic inflammatory phase with elevated levels of cytokines, such as interleukin (IL)-1β, tumour necrosis factor (TNF) and IL-6, the induction of COX-2, and enhanced PGE_2_ production [57]. We used this model to assess the regulation of angiogenesis in chronic inflammatory disease. Our results indicate that ACRH and QRF significantly and dose-dependently reduced the VI associated with FCA-induced inflammation, without affecting granuloma size (Tables 2 and 3). The significant suppression of VI elicited by ACRH and QRF indicates there was a significant reduction in the carmine content in blood vessels surrounding the granulomatous tissue. The lowest dose of QRF (10 mg/kg) reduced angiogenesis to a level similar to that achieved by 10 mg/kg indomethacin. Therefore, we consider that lower doses of QRF might show better potency once the major bioactive compound has been isolated.

ACRH and QRF blocked angiogenesis without markedly reducing granuloma growth. This differs from the effects of glucocorticoids in this model, which severely inhibit granuloma formation [58]. However, our results are consistent with those reported by Gilroy et al. [59], who used the same model to differentiate between COX-1 and COX-2 inhibitors. This explained the reduction in granuloma weight achieved by indomethacin in our study, consistent with the results of aspirin, a selective COX-1 inhibitor, in a prior study [60]. We found that QRF had a greater anti-angiogenic effect in our study, as the effects of 10 mg/kg QRF and 100 mg/kg ACRH were similar. Although the mechanism is not fully understood, we think that ACRH and QRF contain a selective COX-2 inhibitor, considering that our results are consistent with those reported by Gilroy et al. [59].

It is well established that inflammation promotes angiogenesis through several ways. Inflammatory cells such as macrophages, lymphocytes, mast cells and fibroblasts are capable of stimulating vessel growth [60]. Inflammatory mediators, including PGE_1_, PGE_2_, TNF, IL-1, IL-6 and IL-8, in addition to having pro-inflammatory activities, are capable of directly and/or indirectly inducing angiogenesis *in vivo*, which in turn may stimulate tumourigenesis [61].

As inflammation plays a crucial role in inducing rapid angiogenesis in the air pouch model, suppressing inflammation may help to reduce angiogenesis. Our present results may support those of a previous report describing the anti-inflammatory activity of *Ardisia crispa* roots [26]. The primary mode of action of ACRH and QRF might involve anti-inflammatory activities, with their anti-angiogenic potential being secondary. The ability of ACRH and QRF to suppress angiogenesis might be due to their ability to inhibit COX activity. Inflammatory mediators (e.g., PGs) and enzymes (e.g., COX) are involved in rheumatoid arthritis and cancer-induced angiogenesis [3]. Inhibition of COX downregulates the production of inflammatory mediators, and thus reduces the levels of angiogenic mediators [56]. Further studies are needed to confirm the anti-angiogenic activities of ACRH and QRF using a series of models displaying prominent features of angiogenesis, particularly endothelial proliferation, migration and tube formation [62] to further elucidate the mechanisms underlying the anti-angiogenic effects of ACRH and QRF.

## Conclusions

The results of this study indicate that ACRH and QRF exert anti-angiogenic effects by suppressing VEGF-induced permeability and reducing inflammation in adjuvant induced air pouch granuloma in mice *in vivo*. These results confirm the association between angiogenesis and inflammation that was established in previous studies. However, the exact mechanism underlying the anti-angiogenic effects of ACRH and QRF remains to be confirmed. Our current findings provide a scientific basis that will foster further studies in this field.

## Competing interests

The authors declare that they have no competing interest.

## Authors’ contributions

DEZAH performed the research and wrote the manuscript. RAH contributed to the experimental design, data interpretation, editing and submission of the manuscript. LSY contributed to the experimental design and data interpretation. CNMT contributed to the experimental design. YLT performed some of the research and data interpretation. All authors read and approved the final manuscript.

## Pre-publication history

The pre-publication history for this paper can be accessed here:

http://www.biomedcentral.com/1472-6882/13/5/prepub
